# Book Reviews

**Published:** 1915-08

**Authors:** 


### BOOK REVIEWS

Books mentioned may be inspected at and ordered through this office. So far as possible, books received in any month will be reviewed in the issue of the second month following.

**International Medical Annual.** Edited by a staff of British and American physicians and surgeons. Published by Wm. Wood & Co., N. Y., 760 pages, 71 full page plates, $4.

A glossary of new words begins the volume which is arranged alphabetically by comprehensive titles. It is also indexed in detail. This is the 33d year of this cyclopaedia of current medical literature. The topics are often illustrated and are rather full discussions so that the work is not merely an index but a practical guide for the physician.

---

**Annual Report of the Health Officer of the Port of New York** (Joseph J. O'Connell) to the legislature, for the year ending, September 30, 1914.

This is an interesting report, showing the activities of this branch of the health work of the state and including much matter of scientific interest.

---

**Curschmann's Text Book of Nervous Diseases.** Authorized English version by Charles W. Burr, B. S., M. D., Philadelphia. Published by P. Blakiston's Son & Co., Philadelphia. 2 volumes, 1132 pages, 247 illustrations, $12.

This work is written by the following: Aschaffenberg, Curschmann, Kinkelnburg, Gaupp, Hirsch, Jamin, Ibrahim. Curschmann, Finkelnburg, Liepmann, Mueller, Schlesinger, Schoenborn, Starck, Steinert, and the editor. It comprises the following general sections: General Diagnostics, Peripheral Nerves, Spinal Cord, Myopathies, Brain, Organic Nervous Diseases of Childhood, Hyperkinetic Diseases, Psychasthenic States, Sympathetic Nervous System, Vaso-Motor and Trophic Diseases, Intoxications, Operative Treatment, Neurasthenia, Psyasthenia, Hysteria and Borderland Mental States. The monographic method insures personal responsibility and attention to details while the whole has been systematically arranged. It is scarcely necessary to say that the work is comprehensive, and of the highest order.

---

**The Cancer Problem.** Wm. Seaman Bainbridge, A. M., Sc. D., M. D., New York. Published by the Macmillan Co., N. Y., 534 pages, illustrated.

This work is a philosophic study of the subject on broad lines, of interest sociologically as well as medically, it does not aim to describe technic, either of diagnosis or treatment, but it cannot be described as non-technical. All sorts of information bearing on the subject has been collated, with the idea of having a substantial foundation of fact and practical information. For instance, in discussing history, the author alludes briefly to the antiquity of cancer, omits the legendary and superstitious views of cancer, except for an occasional allusion in passing, but summarizes the principal research institutions of the world of which the N. Y. State Institution at Buffalo was the pioneer. Botanic and Zoologic distribution of tumors is reviewed, the general conclusion being that any multicellular animal organism may develop benign and malignant neoplasms. Neither strictly racial immunity nor, conversely any special addiction due to climate, soil, diet and habits of life has been demonstrated. Almost every conceivable practical question as to the nature and ultimate control of cancer is answered in this book, so far as established fact or disproved theory is concerned. That the answer is often incomplete or a confession of ignorance is to be regretted but the ultimate satisfactory answer is hastened by a frank knowledge of where we stand.

---

**1914 Collected Papers of the Mayo Clinic,** Rochester, Minn. Octavo of 814 pages, 349 illustrations. Philadelphia and London: W. B. Saunders Company, 1915. Cloth $5.50 net; Half Morocco $7.00 net.

This is the sixth annual volume. Covering a variety of topics, it is not susceptible of review in detail but several articles dealing with the etiology and pathogenesis of cancer and the studies of the thyroid, are particularly interesting, from a general medical standpoint. The description of a visit to European hospitals, bibliographic tables and suggestions to medical writers are in marked contrast to the technical monographs and perhaps equally valuable.

---

**Alveolodental Pyorrhea.** By Charles C. Bass, M. D., Professor of Experimental Medicine and Foster M. Johns, M. D., Instructor in the Laboratories of Clinical Medicine at the Tulane University Medical College, New Orleans, La. Octavo volume of 167 pages, with 42 illustrations. Philadelphia and London: W. B. Saunders Company, 1915. Cloth, $2.50 net.

This is a comprehensive treatise on an old subject from the new standpoint of amoebic infection. Without in any way opposing this theory, we think that a word of caution is in order. It is scarcely possible that all of the observations on the relation of chemic processes to pyorrhoea are without foundation in fact nor is it definitely established that they can all be explained as direct results of amoebic action. Some regard the endamoeba pyorrhoealis as a harmless parasite and even without accepting this view, the practical importance of various bacteria, saprophytic and pyogenic, should not be set aside without considerable investigation, in the laboratory and the clinic. But, whatever be the results of such experience, the present work is timely and valuable.

**The Treatment of Fractures:** With Notes Upon a Few Common Dislocations. By Charles L. Scudder, M. D., Surgeon to the Massachusetts General Hospital; Associate in Surgery at the Harvard Medical School. Eighth Edition, Revised and enlarged. Octavo volume of 734 pages, with 1057 original illustrations. Philadelphia and London: W. B. Saunders Company, 1915. Polished Buckram, $6.00 net; Half Morocco, $7.50 net.

While the monograph on a phase of medical science developed by modern research has become a type of medical literature of the last quarter of a century the equal importance of systematic treatises on familiar subjects, brought up to date in the light of the same scientific methods plus accrued experience, must not be forgotten. The first copyright on Scudder's work was granted in 1900. There has been much opportunity for advance in fifteen years. What impresses one most forcibly in contrasting this edition with the sections on fractures in the older surgeries, is the degree to which the treatment of fractures has progressed beyond mere bone setting. Not only has the direct attempt to secure fragments of bone in proper place and to stimulate union attained a position of practical utility, far beyond the mere external support of a fractured bone left for repair to purely natural forces, but pathologic fractures have assumed a larger and more hopeful field and the attention to nervous and other lesions incidental to fracture, is greater than formerly. Codman's chapter on the Roentgen rays adds much to the value of the work, his criticism of mistakes in interpretation of skiagraphs being especially worth reading.

---

**Diarrheal, Inflammatory, Obstructive, and Parasitic Diseases of the Gastro-Intestinal Tract.** By Samuel G. Gant, M. D., LL. D., Professor of Diseases of the Colon, Sigmoid Flexure, Rectum, and Anus at the New York Post-Graduate Medical School and Hospital. Octavo of 604 pages, 181 illustrations. Philadelphia and London: W. B. Saunders Company, 1915. Cloth $6.00 net; Half Morocco $7.50 net.

Many of our readers have had the pleasure of hearing Gant speak on certain phases of his work in intestinal diseases. He works, speaks and writes with an animation usually found only in those devoted to a hobby. He subjects theoretic studies to the rigorous test of practical experience. He is bold in his methods of attacking a problem but conservative in selecting the cases to which radical measures are applicable. Diarrhoea can no longer be considered as a disease nor as a symptomatic entity. It is viewed—quite literally—from the standpoint of symptomatology and physical examinations of the ordinary external type, through the speculum, through the laboratory, and even through an incision in the abdominal wall. It is not so much a symptom as an inevitable result of neurotic disturbances, of inflammatory processes, of specific infections and parasitic involvement, of disturbances of physiologic processes more or less intrinsic, of toxic conditions, extrinsic and intrinsic. Its treatment may best be hygienic or medical, even medicinal in the narrow sense, or local so far as the bowel may be reached from without through the anus or some preternatural opening, or surgical in a very direct sense. The author's views are positive but not revolutionary. Originality marks every page of the book, but it is not the originality that neglects general knowledge nor that emphasizes mere personal bias. The work is both scientific and practical. It illuminates a subject about which there is much ignorance and a tendency to superficial consideration. If we could imagine a practitioner to whom the subject would have no direct interest, the book would still be worth reading simply as suggestive of how quite different medical problems should be handled.

---

**Progressive Medicine,** A Quarterly Digest. June, 1915, issue. Edited by Hobart A. Hare and Leighton F. Appleton of Philadelphia. Published by Lea & Febiger. $6 per annum of four volumes.

The present volume contains 445 pages and 100 illustrations. The subjects discussed are : Hernia, Wm. B. Coley; Surgery of the Abdomen, John C. A. Gerster; Gynaecology, John G. Clark; Diseases of Blood, Ductless Glands, etc., Alfred Stengel; Ophthalmology, Edward Jackson. Pyr's method of applying a powerful magnet after ingestion or injection of iron suspensions; Neisser and Brauning's observations on haemokonia, applied by Jeannin and Levant to the test of hepatic function by noting lipaemia microscopically; the review of the pineal body; impress one as particularly interesting and novel, though not more valuable than the careful studies of more familiar topics.

---

**What Every Mother Should Know.** Charles Gilmore Kerley, N. Y. Published by Paul B. Hoeber, N. Y. 107 pages, including blanks for memoranda, illustrated, paper covers, 35 cents.

We are disappointed in this book. From its title, we anticipated a lot of popularized medical science and sociology that would confuse the average mother and endanger her offspring. On the contrary, it is a brief, sensible treatise on feeding, routine care and unofficious first aid. Its title is correct and we heartily recommend it.

---

**Outlines of Internal Medicine.** For the Use of Nurses. By Clifford Bailey Farr, A. M., M. D., Instructor in Medicine, University of Pennsylvania; Assistant Visiting Physician, Philadelphia General Hospital; Pathologist to the Presbyterian Hospital. 12mo., 408 pages, illustrated with 71 engravings and 5 plates. Cloth $2.00 net; Lea & Febiger, Publishers, Philadelphia and New York, 1915.

This book, like others of the series, aims to give the nurse a sufficiently broad knowledge of what lies behind her direct duties to the sick. This purpose is sometimes attacked on account of the well-known danger of a little knowledge— and however thoroughly one studies any subject, his knowledge is always far short of the ultimate total—and particularly because it may tempt nurses to that assumption of medical responsibilities which is so clearly portrayed and usually commended in works of fiction. But this danger is balanced by that of ignorant automatism and it seems to us that the author has, all things considered, chosen a happy mean.

---

**War Surgery.** Edmond Delorme, Medical Instructor General of the French Army. Translated by D. de Meric, Surgeon to the French Hospital, London, published by Paul B. Hoeber, N. Y. 248 pages, illustrated. $1.50.

This is a small compend, exceedingly timely for Europe, happily not for America.

---

**Occupational Affections of the Skin.** R. Prosser White, London. Published by Paul B. Hoeber, N. Y. 165 pages, illustrated, $2.

This work takes up the subject of dermatology from an unusual standpoint. It is a very thorough discussion of the chemic irritants, exposures to cold, immersion of parts and to micro-organisms of various trades. The chapter on poisonous plants is especially interesting. Throughout the work and in a final index, abundant reference to authorities is given. There is so much of new information and of that collated with difficulty from ordinary works on dermatology, that this book should interest not only dermatologists but all physicians.

